# Eating Disorders and Their Associated Risk Factors among Iranian Population – A Community Based Study

**DOI:** 10.5539/gjhs.v5n1p193

**Published:** 2012-12-10

**Authors:** Behshid Garrusi, Mohammad Reza Baneshi

**Affiliations:** 1 Neurosciences Research Center -Kerman Medical Sciences University, Kerman, Iran; 2 Research Center for Modeling in Health, Institute of Futures Studies in Health, Kerman University of Medical Sciences, Kerman, Iran

**Keywords:** eating disorders, contributing factors, Iran

## Abstract

**Backgrounds:**

Many socio cultural variables could be affect eating disorders in Asian countries. In Iran, there are few researches regarding eating disorders and their contributing factors. The aim of this study is to explore frequency of eating disorders and their risk factors in an Iranian population.

**Materials and Methods:**

About 1204 participants were selected aged between fourteen to 55 years. Frequency of eating disorders and effects of variables such as demographic characteristics, Body Mass Index (BMI), use of media, body dissatisfaction, self-esteem, social comparison and social pressure for thinness in individuals with and without eating disorders, were assessed.

**Findings:**

The prevalence of eating disorders was 11.5% that included 0.8% anorexia nervosa, 6.2% full threshold bulimia nervosa, 1.4% sub threshold anorexia nervosa and 30% sub threshold binge eating disorder. Symptoms of bulimic syndrome were greater in males.

**Conclusion:**

In Iran, eating disorders and related problems are new issue that could be mentioned seriously The identification of these disorders and their related contributing factors are necessity of management and preventive programs planning.

## 1. Introduction

Eating disorders that include Anorexia Nervosa (AN), Bulimia Nervosa (BN), Binge Eating Disorder (BED) and Eating Disorders Otherwise Not Specified (EDNOS) are known as disorders with major health consequences (American Psychiatric Association, 2004; Howk & van Hoken, 2003), such as restrictive dieting, depression and even death. Body dissatisfaction and disturbances in body image are regarded as the central cores in eating disorders, and such affected people evaluate themselves as obese, even if they are thin.

Eating disorders were known as a phenomenon of western culture especially amongst women (Howk & van Hoken, 2003); however, over recent years, eating disorders are increasing in eastern cultures as well (Chan & Owens, 2005; Chen & Jackson, 2008; Mellor et al., 2009). The prevalence of eating disorders in western countries was reported to be about 0.3 to 1% amongst young females (Howk &van Hoken, 2003). In Eastern countries, the frequency of eating disorders is different. Nishizawa et al. (2003) suggested that the prevalence of subclinical types of eating disorders are higher than the ones in the clinical setting. Many researchers have found similarities between eating disorders in the west and some Asian cultures such as China (Jackson & Chen, 2007). Eating disorders have been reported about 3 to 10% amongst Chinese females (AM Lee & S Lee, 2000). In the south western region of Asia, there are a few researches about the prevalence of eating disorders; for instance, in United Arab Emirates, 2% of adolescent girls met the diagnostic criteria for eating disorders (Thomas, Khan, & Abdulrahman, 2010). Regarding the eating behavior investment study, 33% of Omani teenagers had vulnerability for expression of anorexic –like behavior and 12.3% for binge eating or bulimia (Al-Adawi et al., 2002). In Jordan, adolescent girls also had bulimia nervosa (0.6%), binge eating disorder (1.8%) and eating disorder not otherwise specified (31%). Although in this study, there was no anorexia nervosa reported (Yousef Mousa, 2010).

Despite increasing attention to the ideal body in recent years in Iran, there are a few researches about body image and consequences such as eating disorders in general population or high risk groups. In one study, the comparison between the body image in Iranian women (living in Iran) and American women (living in US) revealed that both of these two groups had lower body satisfaction when compared to men; however, Iranian women had greater body satisfaction in comparison with American women (Akiba, 1998). In Abdollahi & Mann`s study (2001), there was no difference between body concerns and eating disorder prevalence in Iranian women living in Iran and America. She concluded that exposure to western media and acculturation was not related to body concerns and eating disorder symptoms. Nobakhat & Dezhkam (2000) showed there was a “lifetime prevalence of 0.9% for AN, 3.2% for BN, and 6.6% for the partial syndrome among Iranian schoolgirls”. In another study, the prevalence of eating disorders among Iranian high school girls was about 6.3% with eating disorder symptoms [including 1.7% anorexia nervosa, 1.7% bulimia nervosa and 2.9% kinds of not otherwise specified (NOS)] (ShamsaddineSaeed et al., 2009).

Various variables were known risk factors in developing eating disorders but the role of sociocultural variables in developing eating disorders is very prominent (Howk &van Hoken,2003; Chen & Jackson, 2008; Mellor et al., 2009). Introducing an ideal body, as a need for success and acceptability would elicit one to achieve an ideal body (Ricciardelli & McCabe, 2004; Chan & Owens, 2005; Thomas, Khan, & Abdulrahman, 2010). Thompson et al. (1999) have introduced tripartite influence model of body image disturbances; based on this theory, peers, parents and media are the most important socio-cultural factors that could act as potential risk factors for body image disturbances. These factors would influence body satisfaction via two mechanisms including social comparison and ideal body internalization (Thompson et al., 1999). The family emphasis on beauty, body shape and eating behaviors could result in eating disorders; this aforementioned role was shown in young females (Kluck, 2010). In addition, media plays a great role in body dissatisfaction. Exposures to the western ideal body can change ideal body images via internalizing specific body types in both genders (Thompson et al., 1999; Ricciardelli & McCabe, 2004; Thomas, Khan & Abdulrahman, 2010).

Asian cultures are include many different cultures and considering combining of various ethnic groups under a single “Asian” category obscures important group differences” (Yates, Edman, & Aruguete, 2004). Eating disorder in Iran is a new matter, and there is no sufficient data about it. Therefore this study will aim to identify some of the contributing factors in eating disorders in Iran

## 2. Methods

This study was carried out in Kerman, the capital of the largest province in Iran. This cross-sectional survey was conducted as a part of a larger study “Body satisfaction and related issues in Iran” its method is explained in more detail elsewhere (Garrusi, Garousi, & Baneshi, 2012). This research was approved by Research and Ethical committee of Kerman University of Medical Sciences.

### 2.1 Participants

For estimation approximately 20 regression coefficients (such as demographic variables, body mass index, body dissatisfaction, body steam, comparison with others, social pressure for body change and self steam), at least 200 events were necessary.

We adopted a multistage sampling. First, the city of Kerman was stratified to 10 blocks. From each block, approximately 120 households were selected. In each block, the first household was selected at random. The remainder of the blocks were selected following a systematic sampling approach About 1204 Participants were selected, aged between fourteen to 55 years old. In case no one was at home, the next household was selected. In each household, only one subject was interviewed. We tried to balance the distribution of male and female samples in the interviews.

To do so, interviews were conducted both in the morning and afternoon (Response rate =98%, N=1181). All of participants completed informed consent.

### 2.2 Outcomes

#### 2.2.1 Eating Disorder Diagnostic Scale (EDDS)

The prevalence of eating disorders and their sub types were assessed based on this instrument. This self-reported 22 item questionnaire is based on DSM-IV criteria for eating disorder screening (Anorexia nervosa- Bulimia nervosa –Bing eating disorder). It has been revealed that this questionnaire is sufficiently sensitive to detect eating disorders (Stice, Fisher, & Martinez, 2004). The scale had acceptable internal consistency (Stice, Telch, & Rizvi, 2000), test retest reliability and convergent validity in the Persian version (ShamsaddineSaeed et al., 2009).

### 2.3 Risk Factors

#### 2.3.1 Demographic/Characteristics

We collected demographic characteristics of participants, including gender, age, and marital status (single, married, divorced or widowed). The age was categorized as less than twenty, twenty to forty, and more than forty years old.

#### 2.3.2 Socio-Economic Status

These items included education (illiterate or elementary school, high school, and university degree), occupation, and economic status (low, medium, good, or excellent).

#### 2.3.3 Body Mass Index (BMI)

In addition, weight and height were measured by trained persons using standardized methods (Al-Sendi, Shetty & Musaiger, 2003). To assess the association between BMI level and gender in adults, applying cut-offs at 18.5, 24.9, and 29.9, subjects were categorized into four groups: BMI <18.5 (thin), BMI =18.5-24.9 (normal), BMI =25-29.9 (overweight), BMI >30 (obese) for adults (Al-Sendi, Shetty, & Musaiger, 2003) (only 3.8% of our sample was adolescents aged <19years old. In this group, BMI were calculated based on WHO cut off for children and adolescents).

#### 2.3.4 Media

To evaluate the impact of media on body change strategies, we asked the participants whether they used western television, Iranian television, magazine or internet.

### 2.4 Measures

#### 2.4.1 Body Satisfaction

In this study, we used Figure Rating Scale (Stunkard, Sorensen, & Schulsinger, 1983). This scale has been used for the assessment of body image (Fallon & Rozin, 1985). This scale includes nine silhouettes (male-female), that are numbered from 1 (very thin) to 9 (severely obese). The participants were asked to identify which image was similar to their own current size and shape and also which image they would like to be similar to (ideal). Body dissatisfaction was assessed by subtracting the ideal size from the current size (Fallon & Rozin, 1985). This simple body satisfaction assessment has been used in many studies with acceptable reliability and validity (Fallon & Rozin, 1985). The psychometric properties of Figure Rating Scale has been used in Persian, and their test-retest reliability (α=0.79), convergent validity and internal consistency were approved (α=0.75) (Zanjani & Goodarzi, 2008). Participants were classified into three groups with no body dissatisfaction (if their current and ideal shapes were the same), mild dissatisfaction [when the difference (body dissatisfaction -BD) score was 1], and severe dissatisfaction (when the difference was higher than 1).

#### 2.4.2 Physical Appearance Comparison Scale (PACS)

This questionnaire is a five item likert-type scale instrument that has acceptable reliability and validity for body image assessment (Thompson, Heinberg, & Tantleff, 1991). This scale assesses the tendency of one’s body comparison with others. Answers were scored from never (0) to all times (5). Internal consistency was assessed by chronbach`s alpha coefficient with alpha level of 0.72. In item-scale correlation, we realized that the exclusion of either of the items 1, 2, 3, or 5 would reduce the alpha level. However, the exclusion of item 4 resulted in an increase from 0.72 to 0.84. In addition, the correlation between item 4 and the scale (after correction for overlapping) was as low as 0.02. Therefore, it seemed that item 4 could be excluded. For final checking, factor analysis was done and results showed that item 4 should be put in a separate category. Such results were achieved in other researches as well (Keery et al., 2005; Jackson & Chen, 2007). Therefore, final analysis of PACS was done by 4 items (1, 2, 3, and 5) and it was considered as PACS4. The validity of this questionnaire in Persian was acceptable.

#### 2.4.3 Body-Esteem Scale for Adolescents and Adults (BES-AA)

This self-reported likert-type scale consists of 23 items, which evaluate three subscales: 1-BEA: appearance (general feeling about one’s own), 2-BEW: weight (satisfaction) and 3-BEAT: attribution (judgment of others’ views). The subscales have high internal consistency and 3-month test-retest reliability (Mendelson, Mendelson

& White 2005). Internal consistency and item-scale correlation of Persian version were acceptable. Alpha value was 0.88. The item scale correlation after correction for overlapping ranged from 0.18 to 0.70 (Garrusi, Garousi & Baneshi, 2012).

#### 2.4.4 Perceived Socio-Cultural Pressure Scale (PSPS)

This inventory scale includes ten items that assess the role of friends, media, a dating partner and family on the change of one’s physical appearance. This inventory scale had suitable reliability and validity for the assessment of social pressure on the person for body change (Stice. Bearman, 2001). The Persian version of this questionnaire had acceptable reliability (α=0.92). To assess the convergent validity Rosenberg esteem scale was used. Pearson correlation coefficient was -0.26 with a P-value less than 0.0001 (Garrusi, Garousi, & Baneshi, 2012).

#### 2.4.5 Rosenberg Self-Esteem Scale (RSES)

To evaluate self-esteem, Rosenberg Self-Esteem Scale (RSES) was applied. This questionnaire includes ten items of global statements and is scored from 1 (strongly disagree) to 4 (strongly agree). Negative items are scored negatively. Psychometric properties of this questionnaire in Persian was acceptable (Shapurian, Hojat, & Nayerahmadi, 1987).

### 2.5 Statistical Analysis

Two-sample t and Chi-square tests were applied to compare the continuous and categorical variables between those with and without eating disorders respectively. To identify the variables that influence eating disorders, in a multifactorial setting, logistic regression model was fitted. Results are presented in terms of Odds Ratio (OR) and Confidence Interval (C.I.). All analyses are done using SPSS software 16. P-value less than 0.05 was considered as statistical significance.

## 3. Results

About 54% of the participants were female. The mean (SD) age was 31.06 (10.24) years old with a minimum of fourteen years. Nearly two thirds of the participants were in the middle age group with moderate economic status. Furthermore, about half had university education and were married. The mean (SD) weight of men and women was 69.09 kg (10.83) and 60.30kg (12.58) respectively, resulting in a P-value less than 0.0001. Corresponding figures for height were 173.48 (6.63) and 162.56 (7.7) with a P-value less than 0.0001.

### 3.1 Eating Disorders

There were 133 (11.5%) people who had eating disorders according to EDDS that included anorexia nervosa 9 (0.8%), full threshold bulimia nervosa 73 (6.2%), sub threshold anorexia nervosa 16 (1.4%) and sub threshold being eating disorder 35 (3%). All of the anorexia nervosa and sub threshold anorexia nervosa cases were in the thin group. But there was no thin participant in the other eating disorder subgroups (full threshold bulimia nervosa, and sub threshold being eating).We then compared the proportion of eating disorders among males and females. The results are summarized in Table 1. Information of kind of eating disorder or sex was not available for 23 participants. Therefore, table is organized based on 1181 subjects with complete data.

**Table 1:** Distribution of eating disorders based on gender (n=1181)

Eating disorders	Gender		Total n=1181
	Female 640 (54%))	Male 541 (46%)	
No eating disorder	573(89.5)	475(87.8)	1048(88.7)
Anorexia nervosa	9(1.4)	0	9(0.8)
full threshold bulimia nervosa	26(4.1)	47(8.7)	73(6.2)
sub threshold anorexia nervosa	15(2.3)	1(0.2)	16(1.4)
sub threshold binge eating disorder	17(2.7)	18(3.3)	35(3)

In a series of uni variate analyses, eleven measurements were compared between those with and without eating disorders. We should note that measurements were not available for all participants. Therefore, for each comparison, subjects with available data were analyzed. Mean (SD) for continuous measures, and frequency (percentage) for categorical variables are presented in Table 2. The BMI mean in those with eating disorders was higher than those without (24.43%, 22.64%; p-value = <0.001)). This was the same for PACS4, PSPS, and Body dissatisfaction as well. We have seen that the mean of BEA, BEW, BEAT, and Rosenberg Self-Esteem Scale was significantly lower in those with eating disorders compared to those without (Table 2).

**Table 2:** Mean (±SD) of variables for those with and without eating disorders

Measure	Group				P Value

	Eating Disorder(N=133)		Comparison (N=1048)		

	Mean	SD	Mean	SD	
BMI (Body Mass Index)	24.43	4.60	22.64	3.85	0.001
BEA (General Feeling about One’s Own)	23.55	7.01	27.17	6.06	0.000
BEW (Weight Satisfaction)	20.55	6.28	24.72	6.25	0.000
BEAT (Judgment of Others’ Views)	14.50	4.27	15.78	3.85	0.001
PACS4 (Physical Appearance Comparison Scale)	11.25	4.19	9.76	4.18	0.000
RSES (Rosenberg Self-Esteem Scale)	4.14	4.70	5.99	4.10	0.000
PSPS (Perceived Socio-Cultural Pressure Scale)	21.84	10.65	14.70	6.64	0.000
Body Dissatisfaction	1.29	0.83	0.96	0.88	0.000
Categorical Variables	Frequency	Proportion	Frequency	Proportion	P-Value
Education					
Illiterate or Elementary School	25	15.3%	138	84.7%	0.003
High School	55	14.3%	329	85.7%	
University Degree	53	8.4%	580	91.6%	
Western TV					
Yes	132	11.4%	1021	88.6%	0.56
No	1	6.7%	14	93.3%	
WMagazine					
Yes	89	11.6%	676	88.4%	0.47
No	38	13.2%	249	86.8%	

In a multifactorial logistic regression analysis, with backward variable selection method, we have noted that BMI, BEW, PACS4, PSPS4, and education were independent variables that would influence eating disorders. We have seen than one unit increase in BMI was associated with an eleven percent increase in the risk of eating disorders (Table 3). Furthermore, those with higher education level were at lower risk of eating disorders (P=0.01) (Table 2).

**Table 3:** Identification of variables associated with eating disorders through multifactorial logistic regression analysis

Predictor	Total			Females			Males		

	OR	95%CI	P	OR	95%CI	P	OR	95%CI	P
BMI (Body Mass Index)	1.11	1.06-1.18	0.001	-	-	-	1.50	1.26-1.79	0.000
BEA (General Feeling About One’s Own)	-	-	-	1.06	0.981-1.138	0.14	0.94	0.87-1.01	0.08
BEW (Weight Satisfaction)	0.96	0.91-1.01	0.12	0.95	0.891-1.001	0.05	-	-	-
PACS4 (Physical Appearance Comparison Scale)	1.07	0.99-1.14	0.06	1.09	1.004-1.2	0.04	-	-	-
RSES (Rosenberg Self-Esteem Scale)	-	-	-	0.93	0.86-1.005	0.06	-	-	-
PSPS (Perceived Socio-Cultural Pressure Scale)	1.07	1.04-1.11	<0.001	-	-	-	1.14	1.06-1.23	0.001
Education	0.65	0.46-0.92	0.01	0.43	0.26-0.71	0.001	-	-	-
Body Dissatisfaction	-	-	-	-	-	-	0.41	0.18-0.931	0.03
Western TV	-	-	-	-	-	-	2.42	1.007-5.83	0.048
	Total Correctly Classified:87.3%			Total Correctly Classified:87.6%			Total Correctly Classified:88.9%		

One unit increase in RSES score was associated with 7% decrease in the risk of development of eating disorders with P-value of 0.06

We then stratified our analysis based on gender. We found that variables affecting eating disorders were not the same in male and female participants (Table 3). In particular, we have seen that BMI, PSPS, body dissatisfaction, and watching western TV were only significant in the male group. On the other hand, educational level, SER, PACS4, and BEW were significant only in the females.

Models are fitted in conjunction with backward variable selection method. Therefore, OR is reported only for variable contributed to the final model.

## 4. Discussion

Over recent years, body concerns and desire to be thin have increased in Iran. Many variables do contribute to these changes such as economic development, penetration of western lifestyle, changes in female traditional roles and the effect of media advertisements on ideal body introduction. Therefore, attention to eating disorders, as a consequences of this changes, must be attained. Some of studies have discussed that culture in association with other factors, such as biological factors, influence eating disorders (Ricciardelli & McCabe, 2004; Mellor et al., 2009; Kluck, 2010; Thomas, Khan, & Abdulrahman, 2010). In addition, the symptoms and risk factors of eating disorders may differ in various cultures.

In the present study, the prevalence of eating disorders is about 11.5%; the majority of these individuals had full threshold bulimia nervosa (6.2%) and sub threshold being eating disorder (3%). This prevalence is similar to the prevalence of eating disorders in a Chinese study (Lee & Lee, 2000), and is higher than some other studies in Asian countries (Al-Adawi et al., 2002; Thomas, Khan, & Abdulrahman, 2010).

One reason for such a difference could be due to the application of different instruments or use of clinical investigations in other studies. In a previous similar study in Iran, the prevalence of eating disorders among high school girls was about 6.3% with a limited age and gender group (ShamsaddineSaeed et al., 2009). Our findings showed the prevalence of AN was similar to other studies (Yousef Mousa, 2010), but the prevalence of Bulimia Nervosa and Binge Eating Disorder was higher than some of studies (Mellor et al., 2009). The life time prevalence of eating disorders in females is higher than males (Lee & Lee, 2000; Nobakhat & Dezhkam, 2000).

Some of the studies have revealed that there were differences between the development symptoms and outcomes of eating disorders in both genders (Storvoll, Strandbu, & Wichstrom, 2000; Ricciardelli & McCabe, 2004; Kluck, 2010; Reslan & Saules, 2011).

In this study, the prevalence of full and sub threshold of Anorexia nervosa in females were more than men. Prevalence of full threshold bulimia nervosa was higher in males. Some studies have focused on an increase in eating disorders amongst men, their findings may be due to sociocultural pressures and media influences regarding masculine and strong males’ ideal body and serve binging as a method for achieving this goal (Storvoll, Strandbu, & Wichstrom, 2000). Although eating disorder pattern in Iran could present a need for future investigations, it seems that BN and BED must be mentioned seriously. In addition to the prevalence, contributing factors must be taken into consideration in the eating disorders as well.

Higher BMI was reported as a contributing factor in eating disorders even in binge eating (Stice & Whitenton, 2002). The findings of this study were consistent with other studies. Over recent years, obesity could be due to excessive eating as an eating disorder or it could be a potential risk factor for other eating disorders (Ricciardelli & McCabe, 2004; Neumark-Sztainer et al., 2007). Therefore, it seems that “BMI would contribute to eating disorders via body dissatisfaction” (Stice & Whitenton, 2002). Body dissatisfaction was regarded as a central problem in the development of eating disorders, but in this study, its effect was not obvious in females. This finding may be due to the stronger effects of other variables in developing of eating disorders in females which could result in a decrease in the effects on body dissatisfaction.

Self esteem is identified as a main factor in body perception and evaluations (Mendelson, Mendelson, & White, 2001; Friestad & Rise, 2004). Symptoms of bulimia and attempts at dieting especially recurrent dieting were reported to contribute to low self esteem (Friestad & Rise, 2004). In this study individuals RSES score for female was negatively associated with eating disorders had significant lower self esteem than males. As a predictive factor for eating disorders, this effect was seen in females, that self esteem contribute to eating disorders development negatively. In present study, educational levels had difference between two groups, although many studies were conducted among college students with a high prevalence of eating disorders. This finding may be due to the positive correlation between educational level and self esteem. In Iran, the rate of post graduate education has increased over the past two decades especially in women. An increase in the educational level could lead to a change in social roles which may be due to an increase in self-esteem. Higher self-esteem in educated people could result in lower eating disorders, although this issue necessitates further researches.

Although correlational studies have shown that observing an ideal body in media would cause to increase body dissatisfaction amongst girls (Harrison & Ourselves, 2001; Stice & Whitenton, 2002; Ricciardelli & McCabe, 2004), this correlation in boys has been mentioned only in a few studies (Friestad& Rise, 2004). In this study, although media and western televisions had no effect on eating disorders in the females, such impacts were mentioned as risk factors for eating disorders amongst the males. Jones (2001) believes that girls have more appearance –related social comparisons than boys, but it seems that Iranian males are more influenced by media.

Some studies have argued that a wide distinction between advertised ideal body image in western media and individuals’ cultural norms could decrease the effects of media (Chan & Owens, 2005; Jackson & Chen, 2007; Chen & Jackson, 2008). Iran is a country with Islamic laws. Iranian women wear Hijab, and Iranian men have covered dresses. Demonstrating women and naked men in advertisements is forbidden. Such a difference between western media images and social norms in Iran, especially about women, could lead to these results. The authors of this study recommended further studies for detecting of media effects.

Our findings have shown that an individual comparison with others and perceived pressure from family and peers are the risk factors for eating disorders. In Iranian culture, family plays a prominent role in behavioral control of children, adolescents and even adults. There is a strong relationship among family members, even in married children, and grandparents have a great opinion about offspring and their lifestyles as well. Moreover, in Iranian culture, there is a strong emphasis on social and familial norms and acceptance by others and society is very important too. Amongst Iranian middle social class families, focusing on appearance and beauty has increased. Based on various studies, mothers have greater effects on body dissatisfaction and eating disorders in their daughters (Kichler & Crowther, 2001; Kluck, 2010). The daughters of mother with greater internalization of media messages have greater eating disorders symptoms (Cooley et al., 2008). This effect may be more prominent in Iranian culture since in an Iranian family, child rearing and close relationship with children especially girls stand among mothers’ responsibilities.

Over the past decades, a few researches about eating disorders in Iranian population were done, but there are several strengths in this study. This study was conducted in a general population, both genders and a wide range of age. One limitation of this study was the application of self-reported instruments while more objective measures have more reliability. Changes in Iranian lifestyles especially in recent years, could be result to change of eating disorders prevalence and their socio-cultural contributing factors. Considering This matters and gender differences as a risk factor in eating disorders, such a point should be considered in preventive and therapeutic planning.
